# Long Range Linkage Disequilibrium across the Human Genome

**DOI:** 10.1371/journal.pone.0080754

**Published:** 2013-12-12

**Authors:** Evan Koch, Mickey Ristroph, Mark Kirkpatrick

**Affiliations:** Department of Integrative Biology, University of Texas, Austin, Texas, United States of America; University of Lausanne, Switzerland

## Abstract

Long-range linkage disequilibria (LRLD) between sites that are widely separated on chromosomes may suggest that population admixture, epistatic selection, or other evolutionary forces are at work. We quantified patterns of LRLD on a chromosome-wide level in the YRI population of the HapMap dataset of single nucleotide polymorphisms (SNPs). We calculated the disequilibrium between all pairs of SNPs on each chromosome (a total of >2×10^11^ values) and evaluated significance of overall disequilibrium using randomization. The results show an excess of associations between pairs of distant sites (separated by >0.25 cM) on all of the 22 autosomes. We discuss possible explanations for this observation.

## Introduction

Linkage disequilibrium (LD) is a key feature of genetic variation in human and other populations [1]. Disequilibria between closely-linked sites result largely from random genetic drift or (equivalently) the common ancestry of unrecombined chromosome blocks. These short range disequilibria are of great practical interest. They are the basis for association mapping of genes that contribute to disease and other phenotypes [2]. Blocks of unrecombined chromosome can also be exploited to identify recent and ongoing selective sweeps [3], [4]. While these “long range haplotypes” can extend over a few hundred kb in unrelated humans [5], they still span only a very small fraction of an entire chromosome.

Considerably less attention has been paid to patterns of LD between pairs of sites that are separated by much greater genetic distances (say, 1 cM or more). Since recombination tends to break down disequilibria rapidly between such sites, finding substantial long range linkage disequilibrium (LRLD) suggests that countervailing forces are at work. One possibility is population admixture [6], which has been proposed to explain unusual patterns of LRLD in some human populations (e.g. [7]). A second contributing force is drift. Even in a population at demographic equilibrium, recombination between distant chromosome blocks will largely but not completely erase LD caused by drift. Disequilibria can be amplified by demographic change [8]. Recurrent bottlenecks are particularly effective at generating LD [9], and may have contributed importantly to disequilibria in non-African populations of humans (e.g. [10]). Third, epistatic selection can maintain linkage disequilibrium indefinitely [11]. Epistasis has been implicated in the LD observed between two pairs of genes in humans [12], [13]. Fourth, the hitchhiking of linked sites with a positively-selected mutation can generate large haplotype blocks that result in disequilibria over the region that they span [3], [4]. Fifth, structural variation in chromosomes, such as inversions, can alter patterns of recombination and consequently cause LD to extend over unusually large regions of a chromosome [14]–[16]. Last, certain types of error in the reference genome or the data could lead to the appearance of LD in a population that in fact has none.

Despite the evident interest in these processes, to our knowledge there has been only one previous survey of associations between chromosomal regions across the entire human genome using high-density data. Sved [17] studied correlations in heterozygosity between chromosome blocks. His analysis of the HapMap phase 3 data found evidence of associations between blocks at distances of up to 10 cM and weak correlations between blocks on different chromosomes, but he did not attempt to assess their statistical significance. Sved pointed out that his approach has the advantage that it can use unphased data, but also that it loses power to detect linkage disequilibrium when phased data are available. Lawrence et al. [18] provided a web-based tool for exploring long distance linkage disequilibria in the HapMap data, but did not go on to study patterns in the data. Other studies of LRLD in humans and other species have been restricted to much lower density data [19], [20], to inbred strains [21], and to admixed populations [22].

This paper investigates patterns of LRLD in the YRI population (the Yoruba in Ibadan, Nigeria) from the HapMap Phase 2 dataset of single nucleotide polymorphisms [23]. We chose to focus on YRI because it is the most genetically variable of the three populations in the dataset. YRI also has weaker short-range disequilibria that might otherwise obscure the patterns of LRLD of interest here; indeed, LD decays more rapidly over short genomic distances in this population than any of 52 populations studied by Conrad et al. [24]. We calculated the disequilibria between all pairs of SNPs on the same chromosome, then analyze these data with new statistical methods.

Our goal is to determine if there is a excess of long range linkage disequilibrium that cannot be explained by sampling. Our approach is based on new statistics that summarize the distribution of LD across an entire chromosome. Using null distributions generated by randomization, we find significant excess of disequilibria on all 22 autosomes in the Yoruba population. While finding the specific pairs of chromosome segements that are in strong disequilibrium is not the main goal, as a biproduct of the analysis we do identify candidate pairs. Many of these are at much greater distances than those that have been previously characterized. We discuss several hypotheses that might account for these patterns, but are not able to distinguish between them with our approach.

## Methods

We analyzed the 120 YRI haplotypes that were genotyped at over 2.8×10^6^ SNPs in HapMap Phase 2 (data build 22) [23]. The data are of very high quality in several respects. The genotyping error rate is less than 0.5% [25]. Key to our analyses is that the data are phased haplotypes of entire chromosomes; the phasing is based on parent-offspring trios and has an error rate of only 0.16% [23], [26]. The linkage map for the SNPs was constructed from the YRI sample using a coalescent method [23], [27].

Analyzing these data for LRLD raises four statistical issues: how to measure LD, how to identify pairs of chromosome blocks that are in LRLD, how to quantify chromosome-wide patterns of disequilibria, and finally how to test for the significance of those patterns. The following sections describe the statistical measures and algorithms that we propose for those purposes. These have been implemented in C and Python, and the code is available for download at: http://www.sbs.utexas.edu/kirkpatrick_lab/K/Software.html.

### Measuring disequilibria

We want to distinguish disequilibria in the population from chance associations that result from sampling. Most commonly used measures of linkage disequilibria are not well suited for that purpose [8]. For example, a large value of *D*′ is likely to result from sampling if allele frequencies are near 0 or 1, while even a small value is unlikely to appear by chance if allele frequencies are intermediate and the sample size is large. We therefore use the probability that a value of the disequilibrium *D* as large or larger than that in the sample would be observed if there is no association in the population from which the sample is drawn, conditioned on the sampled allele frequencies at the two loci. This probability, which we denote *p_D_*, is given by the tail of Fisher's exact test [8], [28], [29]. As the distance between a pair of sites on a chromosome grows large (specifically, the product of the recombination rate and the effective population size becomes much greater than 1), the sampling distribution for two-locus haplotypes converges on that of Fisher's exact test [30], [31]. Thus *p_D_* is an appropriate statistic for detecting nonrandom disequilibria in the population. Note that smaller, not larger, values of *p_D_* represent stronger evidence of disequilibria.

### Identifying patches of LRLD

When a pair of distant sites are in disequilibrium, it is likely that other sites near to them will also be associated as a result of short-range associations [17], [32]. In effect, the underlying structure in the data is disequilibrium between pairs of chromosomal blocks rather than between pairs of individuals sites. To control for this effect, we used a simple and efficient *ad hoc* strategy that identifies “patches” of disequilibria.

**Figure 1:**
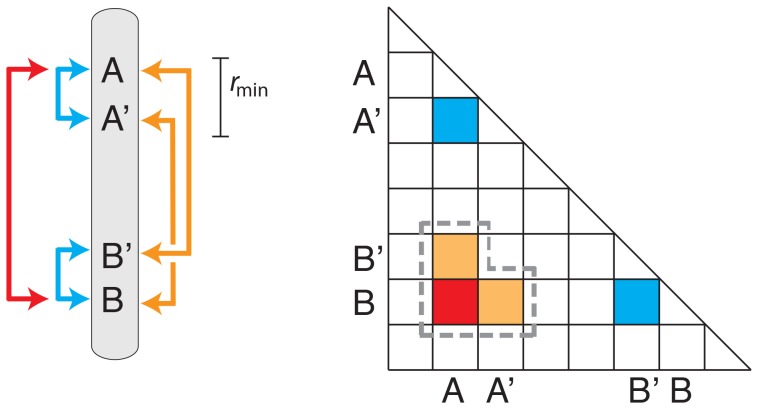
Schematic of the structure of long range linkage disequilibria. *Right:* Sites *A* and *B* are the target of selection or other force that generates disequilibria between them (red arrow). Site *A* is in disequilibrium with site *A′* nearby as the result of shared ancestry of that small segment of chromosome; likewise *B* and *B*′ also show short range disequilibrium (blue arrows). *Right:* A triangle plot of LD. Disequilibria between nearby sites appear as the band along the diagonal. The dash line encloses a “patch” consisting of pairs of widely-separated SNPs that are in LD.

The situation can be visualized using a triangle plot of the type introduced by Miyashita and Langley [33]. A schematic example is shown in Figure 1. There is a pair of distant sites *A* and *B*, a site *A′* near to *A*, and a site *B′* near to *B*. Sites *A* and *B* are the targets of some force (e.g. epistatic selection) that generates disequilibrium between them. Sites *A* and *A′* are in LD as the result of shared ancestry, as are sites *B* and *B′*. These short range disequilibria can lead to secondary long range disequilibria, for example between sites *A* and *B′*, and between sites *A′* and *B*. The result is that regions surrounding sites *A* and *B* will also be in disequilibrium. Visualized on the triangle plot, the two sets of sites involved appear as a patch of LRLD (Figure 1). We therefore aggregate pairs of sites into patches that represent pairs of chromosome blocks that are in LRLD.

We implemented this aggregation using an *ad hoc* algorithm with three steps. First, we calculated *p_D_* between all pairs of SNPs on each chromosome that are separated by more than *r*_min_ = 0.25 cM (corresponding on average to about 250 kb). Pairs of sites linked more tightly than this value were excluded in order to filter out short range disequilibria from the analyses. The value of *r*_min_ = 0.25 cM is greater than the more than 90% of the haplotype blocks (tracts of short-range disequilibria) (Suppl. Fig. 9b in [25]). The effective population size of the YRI population is estimated to be 7,500 [34], so 0.25 cM corresponds to ρ = 4*N_e_r* = 75. That value is expected on theoretical grounds to provide enough recombination to very largely eliminate disequilibrium from shared ancestry [31]. Recombination rates between pairs of sites were calculated from the HapMap linkage map using Kosambi's [35] mapping function.

Second, we identify all pairs of sites whose value of *p_D_* falls below a threshold value that we denote 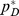. (Recall that smaller values of *p_D_* correspond to stronger evidence for LRLD.) In the third step, we form patches by aggregating these pairs with extreme values of *p_D_* into “patches” of LRLD. Consider two pairs of sites both of which have extreme values of *p_D_*. The sites in the first pair are at positions *A* and *B*, and the second pair at *A′* and *B′*. These two pairs are merged into a single patch if the distance between *A* and *A′* and the distance between *B* and *B*′ are both less than *r*_min_ (see Figure 1). Additional pairs are added to the patch if they meet this distance criterion for any of the pairs of sites already in the patch. Growth of the patch stops when no more pairs of sites that are adjacent to a patch exceed the 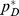 threshold.

The number of patches formed by this algorithm depends on the threshold value 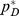 (as well as *r*_min_). We chose 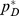 adaptively such that approximately *n*_1_ patches resulted. For Chromosome 1, we used a value of *n*_1_ = 250. For the remaining chromosomes, we chose *p_D_* such there were 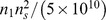 patches, where *n*_S_ is the number of SNPs on that chromosome. All else equal, this rule will generate the same density of patches (that is, patches per pair of SNPs) on each chromosome, which is desirable as it allows us to compare chromosomes. We chose *n*_1_ = 250 because smaller values lead to very few patches on the smallest chromosome (less than 7 on Chromosome 22), and larger values lare computationally expensive.

This algorithm depends on the two nuisance parameters, *r*_min_ and *n*_1_. We return to the choice of values for those parameters below.

### Quantifying patterns of LRLD

We take two approaches to search for nonrandom patterns of LRLD. We first ask whether observed values of *p_D_* are more extreme than expected. For this purpose we determined the most extreme (that is, smallest) value of *p_D_* in each patch, then calculated the mean of these extreme values across all patches on a chromosome. We refer to this statistic as *p_D_* ^max^. Second, we ask whether the number of LRLD patches observed for a given chromosome is greater than expected by chance. We denote this statistic as *n*_P_.

**Figure 2:**
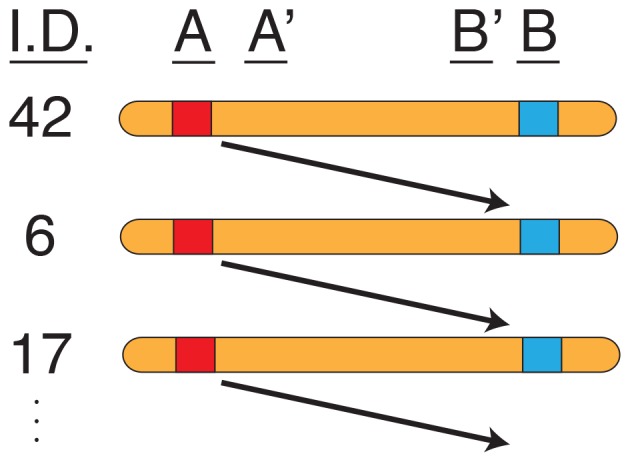
Schematic of the randomization method used to construct null distributions for patterns of LRLD. For each randomization, the identity numbers of the haplotypes are permuted randomly (left hand column). The disequilibrium between sites *A* and *B* is calculated using the allele at site *A* from one chromosome and the allele at site *B* from the following chromosome.

To test for the statistical significance of *p_D_* ^max^ and *n*_P_, we generate their null distributions using a randomization method that is shown schematically in Figure 2. For a given chromosome, the identifiers for the 120 haplotypes are randomly permuted. The disequilibrium between two sites, say *A* and *B*, is then calculated by constructing 120 artificial haplotypes consisting of the allele at site *A* at one haplotype and the allele *B* found in the following haplotype on the permuted list. The values of *p_D_* for all pairs of sites on the chromosome are computed for that permutation. These data are then used to calculate *p_D_* ^max^ and *n*_P_. We constructed null distributions for these two statistics using 1,000 random permutations for each chromosome.

There are two motivations behind this method. First, it preserves the allele frequencies at each site. Second, it maintains the structure of short range disequilibria in the sample. Thus if by chance sites *A* and *B* have an extreme value of *p_D_*, then it is likely that sites *A* and *B*′, and sites *A*′ and *B*, will also (see Figure 1). Consequently, this randomization algorithm produces patches of LRLD that mimic those in the real data but that arise by sampling.

Constructing these null distributions is the most computationally intensive part of our method. For the analyses reported below, over 4.8×10^14^ values of *p_D_* were computed, and the project consumed about 34,000 hours of CPU time. The computations were accelerated by using a lookup table for logarithms of factorials so that −ln(*p_D_*) could be computed solely by addition for any given configuration of genotypes at a pair of SNPs.

### Correlations of LRLD with genomic targets of selection

One possible cause of LRLD is selection. We therefore wanted to determine if the patches of LRLD identified by our algorithm are associated with genome regions that have been previously identified as targets of positive selection. For this purpose we used the catalogue of 722 chromosome regions compiled in Akey's [36] review of genome scans for positive selection in humans, and asked whether they tend to fall in patches of LRLD. These regions were compiled from eight studies that used site frequency spectra or linkage disequilibrium to test for selection (Supplemental Table 1 in [36]). For each patch, we identified the pair of sites associated with the most extreme value of *p_D_*. Next, we counted the number of times that one or both of those sites fall in a region that appears in the Akey catalog. To determine if the number of these occurrences is larger than expected by chance, we again used a randomization method to construct null distributions. Regions of equal length and number as those compiled by Akey were dropped at random onto their respective chromosomes. For each randomization, the number of extreme pairs that had one or both sites within a region were counted and compared with the observed pattern.

**Table 1:** Results for the test statistics *p_D_* ^max^ and *n*_P_.

	*p_D_* ^max^		*n*_P_	
Ch	Value	*p*	Value	*p*
1	1.5×10^−9^	<10^−3^*	247	<10^−3^*
2	5.2×10^−9^	<10^−3^*	316	<10^−3^*
3	1.5×10^−8^	<10^−3^*	186	**0.005**
4	7.7×10^−9^	<10^−3^*	167	<10^−3^*
5	2.3×10^−8^	<10^−3^*	172	0.001*
6	9.9×10^−9^	<10^−3^*	199	<10^−3^*
7	2.1×10^−13^	<10^−3^*	124	<10^−3^*
8	9.3×10^−9^	<10^−3^*	138	<10^−3^*
9	2.5×10^−9^	<10^−3^*	92	0.001*
10	8.6×10^−10^	<10^−3^*	121	<10^−3^*
11	9.3×10^−9^	<10^−3^*	104	<10^−3^*
12	1.9×10^−8^	<10^−3^*	96	<10^−3^*
13	2.4×10^−9^	<10^−3^*	71	<10^−3^*
14	2.4×10^−8^	<10^−3^*	44	0.768
15	9.1×10^−12^	<10^−3^*	33	<10^−3^*
16	2.1×10^−10^	<10^−3^*	33	0.001*
17	6.5×10^−10^	<10^−3^*	21	<10^−3^*
18	5.1×10^−10^	<10^−3^*	41	0.001*
19	7.8×10^−11^	<10^−3^*	8	0.275
20	2.3×10^−10^	<10^−3^*	25	<10^−3^*
21	2.0×10^−10^	<10^−3^*	7	0.05
22	9.8×10^−22^	<10^−3^*	7	<10^−3^*

*p* values give the significance for individual chromosomes (under a one-tailed test). Values in bold are significant at the 0.05 level, and those with an asterisk are significant after a Bonferroni correction for tests of multiple chromosomes. The

## Results

The values of *p_D_* ^max^ and *n*_P_, and their significance levels, are shown for each autosome in Table 1. All of the 22 chromosomes show significant values for *p_D_* ^max^ at the *p*<0.05 level, and all remain significant after a Bonferroni correction for multiple tests. For the second test statistic, *n*_P_, 19 chromosomes show significant values, 18 of which remain significant after the Bonferroni correction. These results suggest there is long-range linkage disequilibrium in the YRI population.

**Figure 3:**
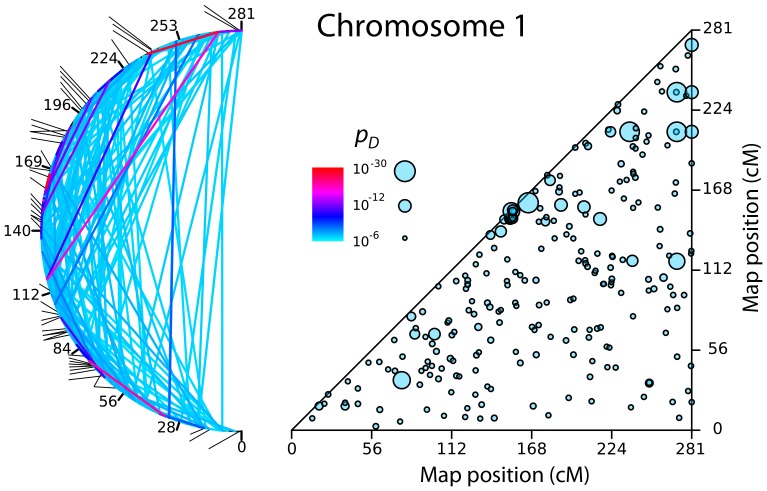
Patches of LRLD on Chromosome 1. *Left:* The circle plot represents patches by lines that connect the chromosome blocks involved. The most extreme value of *p_D_* in each patch is represented by the color of the segment. The line segments on the outside of the circle show regions identified in Akey's [36] catalogue of genomic targets of positive selection. *Right:* The triangle plot shows the patches as circles whose size is scaled to the value of the most extreme value of *p_D_* in that patch.

**Figure 4:**
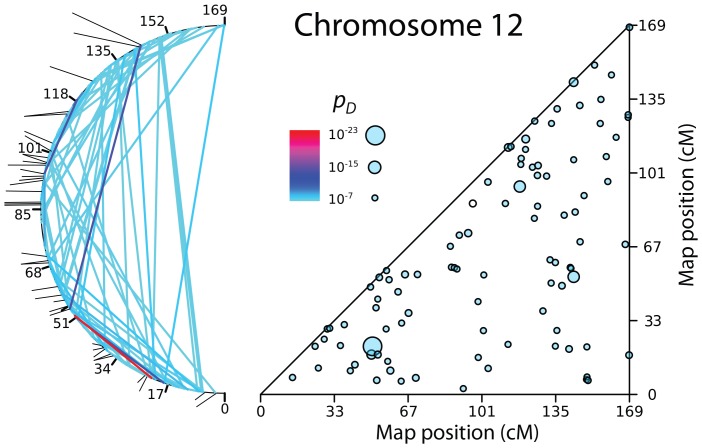
Patches of LRLD on Chromosome 12.

**Figure 5:**
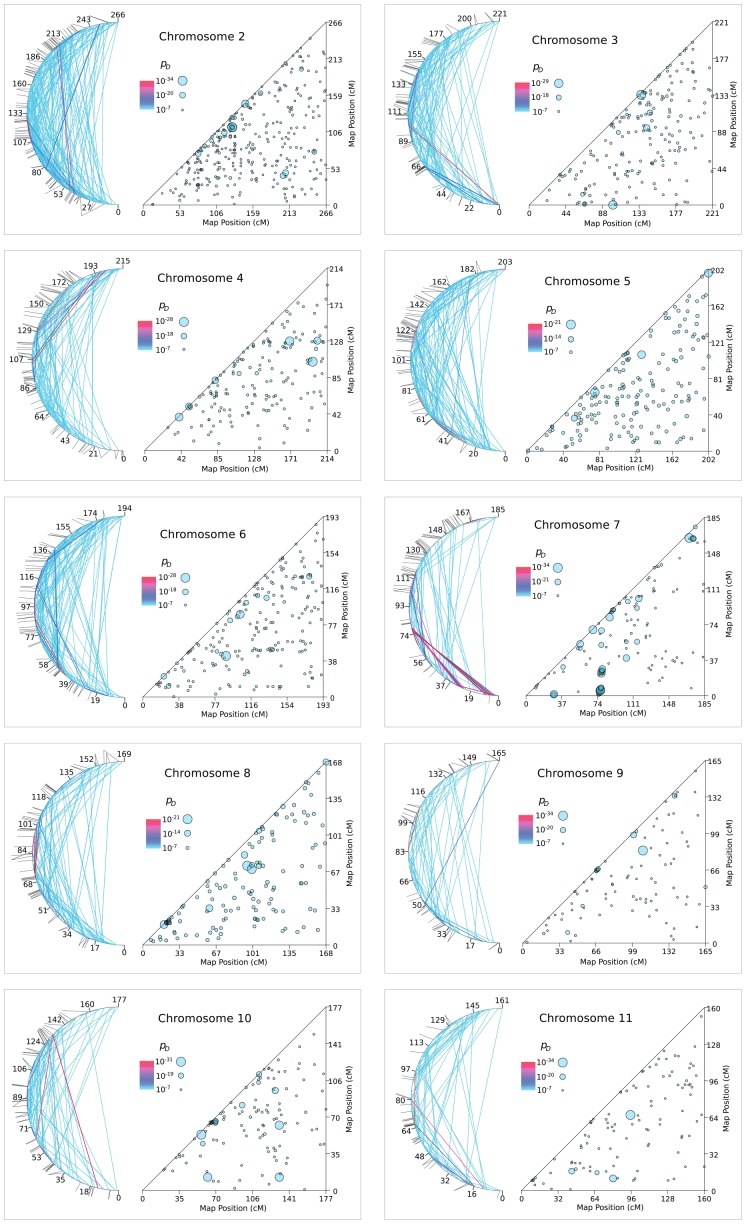
Patches of LRLD on autosomes 2 to 11.

**Figure 6:**
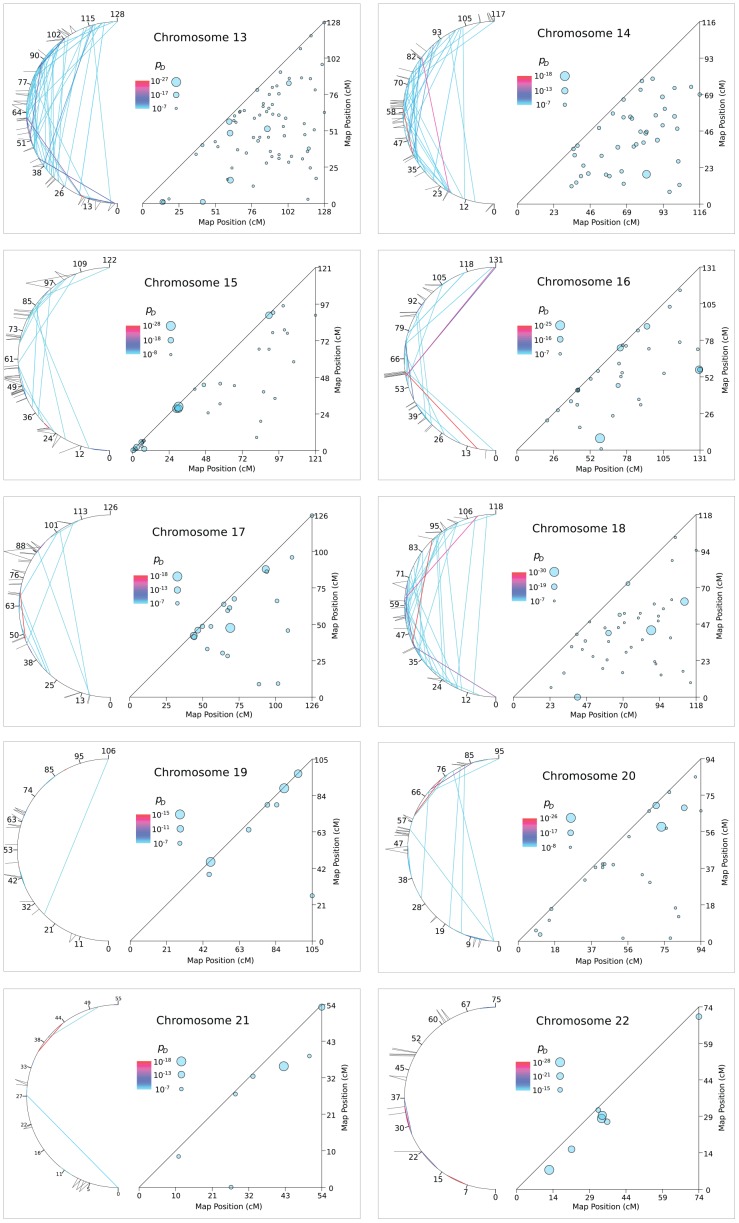
Patches of LRLD on Chromosome 12 to 22.

Figures 3 and 4 show the patches of LRLD on Chromosome 1, which is the largest chromosome, and Chromosome 12, which is intermediate in size. Two representations are shown for each chromosome. The first is a triangle plot of the kind seen in Figure 1. Patches are shown as circles, with radii that are scaled to the maximum value of *p*_D_ for that patch. The second representation bends the chromosome into a semicircle, and connects the two correlated blocks corresponding to a patch with lines whose colors are scaled to *p_D_*. The distribution of patches shown in Figures 3 and 4 are typical for most chromosomes. Exceptions are Chromosome 7, which shows a clumped distribution of patches, and the smallest chromosomes (Chromosomes 17–22), which have a low density of patches because they have a lower density of SNPs per cM. (SNPs in the HapMap dataset are distributed with approximately equal density per Mb, and smaller chromosomes have higher recombination rates per Mb.) Figures 5 and 6 show the corresponding distribution of patches on the other 20 autosomes.

The correlations between patches of LRLD and Akey regions of positive selection are summarized in Table 2. Recall that we considered two possibilities, that either one or both sites in LRLD are found in Akey region(s). At a nominal significance value of *p*<0.05, Chromosomes 3, 5, and 13 show a significant correlation for both sites of a pair, while Chromosomes 6 and 16 show a significant correlation for one site of a pair. After a Bonferroni correction is applied, however, none of these correlations remain significant.

**Table 2:** The number of patches that appear in the Akey's [36] catalogue of putative targets of selection.

	Akey 1		Akey 2	
Chr	*N*	*p*	*N*	*p*
1	52	0.098	2	0.61
2	121	0.12	18	0.52
3	34	0.25	7	**0.0029**
4	37	0.88	5	0.56
5	33	0.57	7	**0.016**
6	42	**0.0064**	3	0.067
7	23	0.52	2	0.51
8	45	0.095	11	0.060
9	12	0.26	2	0.053
10	25	0.43	3	0.27
11	14	0.13	0	0.64
12	16	0.078	0	0.67
13	12	0.95	6	**0.0069**
14	7	0.35	1	0.12
15	7	0.62	2	0.46
16	7	**0.019**	0	0.55
17	4	0.28	1	0.18
18	9	0.10	0	0.66
19	4	0.36	0	0.60
20	2	0.78	0	0.61
21	0	0.78	0	0.53
22	1	0.20	0	0.50

*p* values give the significance for individual chromosomes tested individually. Values significant at the 0.05 level are in bold; none is significant after a Bonferroni correction for tests of multiple chromosomes. Akey 1 gives the number of patches in which one of the two participating sites is in the Akey catalogue; Akey 2 is the number in which both sites do. The

Our algorithm for constructing null distributions of *p_D_* ^max^ and *n*_P_ depend on two nuisance parameters. The first is *r*_min_, which determines what pairs of sites are excluded from the analysis because they are too closely linked, and also determines which pairs of sites are aggregated into patches. The analyses used *r*_min_ = 0.25 cM, and it is possible that this value is too small and some patches are short-range disequilibria resulting from haplotype blocks. The second nuisance parameter is *n*_1_, which sets the density and therefore the number of patches of LRLD that are identified on each chromosome. We initially chose *n*_1_ = 250 as a minimum value, and it is possible that the results will change if it is increased.

Table 3 shows how the results depend on the values of those two parameters. Increasing *r*_min_ up to 0.5 cM and increasing *n*_1_ up to 500 has little effect on which chromosomes show significant values for *p_D_* ^max^. The results also show that the *n*_P_ statistic is less robust. For example, the number of chromosomes showing an excess number of patches declines by almost half as *r*_min_ is varied from 0.25 to 0.5 cM. On the other hand, the number of chromosomes with significant values for *n*_P_ increases with *n*_1_, further indicating that our choice for the density of patches is somewhat conservative. We conclude that the values used for the nuisance parameters (*r*_min_ = 0.25 and *n*_1_ = 250) are reasonable when *p_D_* ^max^ is used to quantify LRLD. The number of patches, *n*_P_, is however much more sensitive to the choice of those values. The choice of *n*_1_ = 250 in particular might have been problematic, as unlike *r*_min_ it is not based on population genetic data. However, Table 3 shows that, at least for *r*_min_ = 0.25, doubling the density of patches only alters the significance of one chromosome in both measures.

**Table 3:** The effects of the values of the nuisance parameters *r*_min_ and *n*_1_ on the number of chromosomes that show significant values for the test statistics *p_D_* ^max^ and *n*_P_ at the nominal *p*<0.05 level (without a Bonferroni correction).

*r*_min_	*n*_1_	*p_D_* ^max^	*n*_P_
0.25	250	22	18
″	375	21	18
″	500	21	19
0.375	250	21	13
″	375	21	14
″	500	20	16
0.5	250	21	10
″	375	21	12
″	500	20	15

**Figure 7:**
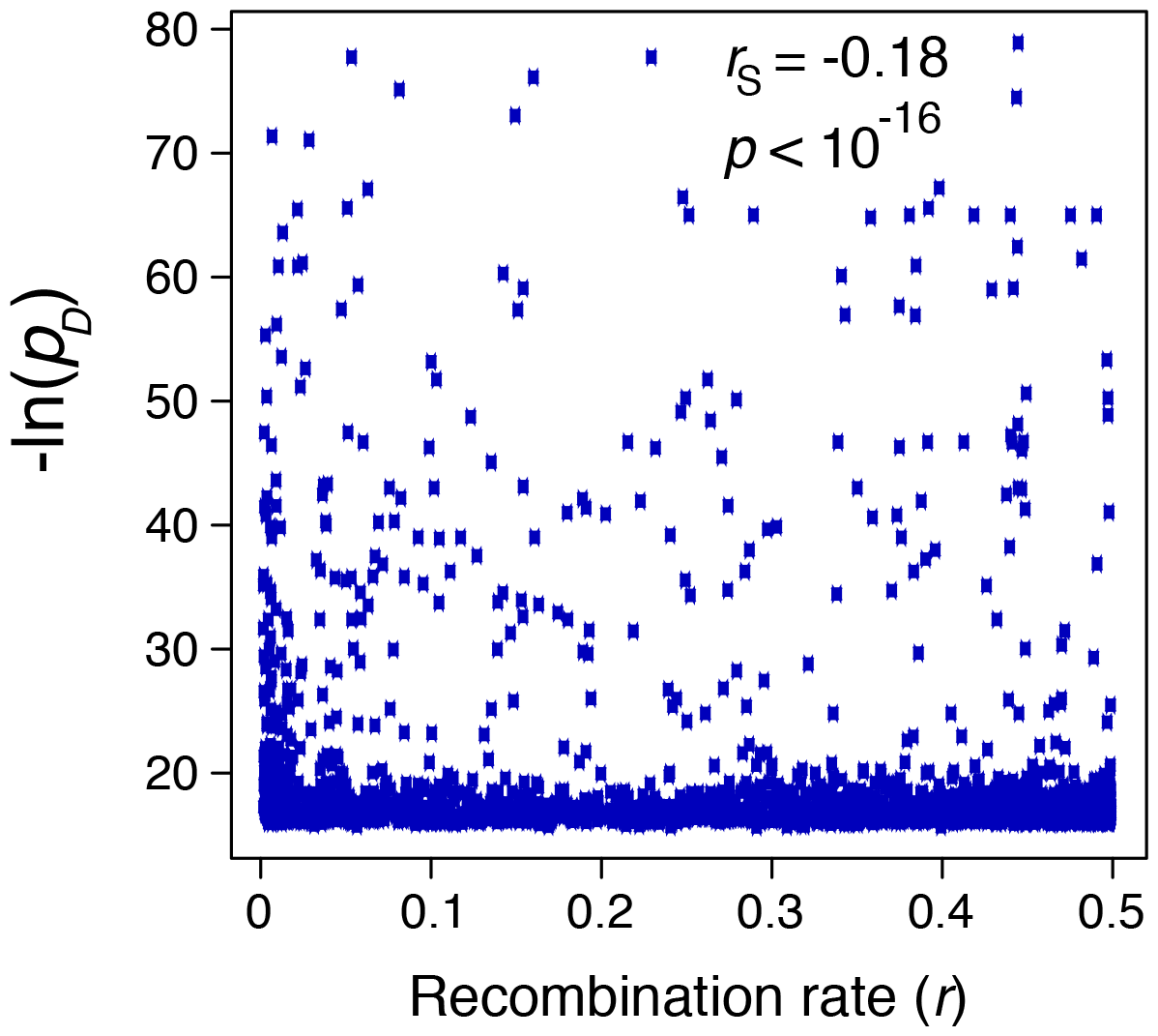
Scatter plot of the maximum value of −ln(p_D_) in every patch across all chromosomes vs the distance r between the patches. Recombination is expected to make this correlation negative.

Because recombination erodes LD, we expect the frequency and strength of LRLD to decline with distance between pairs of sites. We therefore asked how the maximum value of *p_D_* in each patch and the density of patches correlate with distance (measured by the recombination rate). To calculate the density of patches, we grouped patches into twenty bins corresponding to ranges of recombination values, then normalized the number of patches in each bin by the total number of pairs of SNPs falling in that range. Significance was evaluated using Spearman's rank correlation. The results are presented in Table 4. Correlations between *p_D_* and distance are significant (at *p*<0.05) for 11 chromosomes, two of which remain significant after a Bonferroni correction. Figure 7 shows these data pooled across all chromosomes. The correlation is weakly negative (*R_S_* = −0.18) but highly significant (*p*<10^−16^) because the number of points is so large. (The log transformation of *p_D_* used in Figure 7 makes the distribution easier to visualize but does not affect the value of *R_S_*, which is a nonparametric statistic that depends only on the rank order of the observations.)

**Table 4:** Spearman rank correlations between −ln(*p_D_*) and distance, and between patch density and distance.

	−ln(*p_D_*)		Density	
Chr	*r*_S_	*p*	*r*_S_	*p*
1	−0.219	<0.001*	−0.472	**0.037**
2	−0.060	0.290	−0.573	**0.009**
3	−0.155	**0.036**	−0.359	0.120
4	0.029	0.708	−0.426	0.063
5	−0.164	**0.032**	−0.495	**0.028**
6	−0.002	0.976	−0.298	0.214
7	−0.060	0.511	−0.474	**0.036**
8	−0.256	**0.003**	−0.394	0.087
9	−0.239	**0.023**	−0.268	0.265
10	−0.234	**0.010**	−0.519	**0.024**
11	−0.228	**0.022**	−0.311	0.181
12	−0.154	0.135	−0.453	**0.047**
13	−0.117	0.330	−0.214	0.377
14	0.049	0.755	−0.267	0.299
15	−0.634	<0.001*	−0.797	0.002*
16	−0.217	0.232	−0.533	0.064
17	−0.508	**0.024**	−0.797	0.002*
18	−0.369	**0.020**	0.085	0.755
19	−0.607	0.167	−0.191	0.461
20	−0.477	**0.018**	−0.636	**0.030**
21	−0.429	0.419	0.000	1.000
22	0.257	0.658	0.400	0.750

*p* values in bold are less than 0.05; values with asterisks are significant after a Bonferroni correction. Recombination is expected to make −ln(*p_D_*) decline with distance, giving a negative correlation. The

The correlations between patch density and distance are significant for nine chromosomes, two of which remain significant after a Bonferroni correction. These trends seem to be driven by somewhat higher LD between the closest sites as all the correlations become nonsignificant if we exclude pairs of sites separated by less than 10 cM.

**Figure 8:**
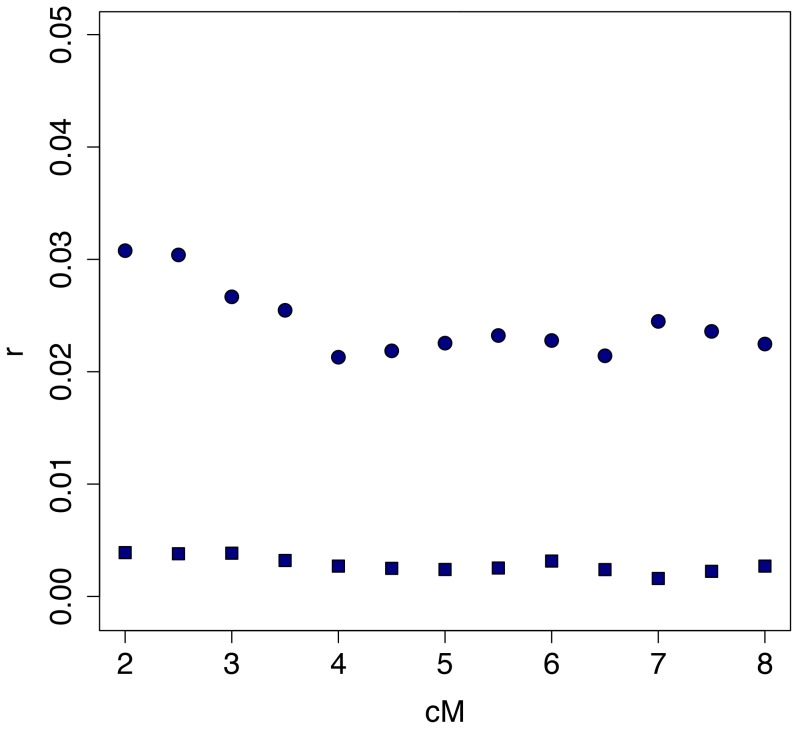
Sved's [17] measure for the correlation of heterozygosity between blocks of 50 SNPs as a function of the recombination rate between the blocks. The squares show the genome-wide average for the Yoruba population (calculated by Sved). The circles pertain to the 10 cM region of Chromosome 8 that has the highest density of LRLD patches identified by our method. The difference is highly significant (*p*<10^−3^).

The only other analysis of associations across the entire genome in the HapMap dataset is that of Sved [17], who found correlations in heterozygosity between regions up to 10 cM apart. We asked if genomic regions that show excess LRLD also tend to show high correlations of heterozygosity. For this purpose, we focused on the 10 cM region of the entire genome that has the highest density of patches identified by our method: the region between 16.5 and 26.5 cM on Chromosome 8. Following Sved's protocol, we calculated the correlation in heterozygosity between each pair of nonoverlapping blocks containing 50 SNPs, and plotted those correlations as a function of the distance (in cM) between the two blocks. Figure 8 shows that the correlations in this region are much stronger than the genome-wide average. This result suggests that chromosomal regions that are outliers by Sved's measure (the correlation in heterozygosity) are also unusual by our measure (the density of LRLD patched). The concordance between the two approaches might indicate there are regions of the genome that have unusual biological properties (e.g. as targets of selection). Another possibility is that the explanation is lies in a statistical artifact, for example highly polymorphic regions may be more likely to appear as outliers by both measures.

One possible source of LRLD is the inclusion of related individuals in the sample. The HapMap sample from the YRI population includes three pairs of related individuals (Suppl. Mat. 5 in [23]). To determine if these relatives might be responsible for the patterns of LRLD we detected, we removed one individual from each of the three pairs and reran the analyses for Chromosome 1. The results are largely unchanged: *p_D_* ^max^ and *n*_P_ remain significant, while few patches change position. We therefore conclude that the close relatives in the YRI sample are not responsible for the patterns of LRLD we see.

Chromosome inversions result in LD between regions near their breakpoints [14], [16]. To assess whether inversions contribute to LRLD in the YRI data set, we asked whether the sites forming our patches fell within approximately 0.25 cM of breakpoints of polymorphic inversions that are identified in two databases. The first is the Database of Genomic Variants [37], which provides a list of inversions that have been verified experimentally. Only seven of the 2,230 patches we found have both sites within 0.25 cM of both breakpoints in that list, and none of those seven had a *p_D_* value within the 10% of the most extreme *p_D_* values across the genome. The second database is of 71 inversions in the YRI population predicted by computational methods [14]. No pairs of sites forming our patches correspond with both predicted breakpoints in this dataset. We therefore conclude that polymorphic inversions contribute little if anything to the patterns of LRLD we see.

In summary, there is evidence for long range linkage disequilibrium across all 22 autosomes in the YRI population. This conclusion is most strongly supported by the *p_D_* ^max^ statistic.

## Discussion

Linkage disequilibrium is classically viewed as an association between pairs of sites or loci. In fact, the underlying structures of these associations involve pairs of chromosome blocks rather than sites [17], [32]. One basic feature of genetic variation in human populations seems to be the existence of associations between pairs of blocks that are separated by large intervening chromosome regions. Although these long-range linkage disequilibria (LRLD) have been little studied, they may be indicators of important evolutionary processes. Detecting and quantifying LRLD from high density genomic data is an open challenge for evolutionary genetics.

Towards that end, here we propose *ad hoc* statistical methods to detect LRLD. Our basic measure of association is *p_D_*, defined as the probability that the observed linkage disequilibrium would be observed by chance if the true disequilibrium in the population is 0. We use *p_D_* as the basis for two test statistics that quantify chromosome-wide patterns of disequilibrium. The first is *p_D_* ^max^, defined as the chromosome-wide average across “patches” of disequilibria of the most extreme value of *p_D_* in each patch. The second is *n*_P_, the number of patches of disequilibria on the chromosome. To test for the significance of *p_D_* ^max^ and *n*_P_, we developed a randomization algorithm that accounts for the effects of short-range disequilibria that are not the focus of our interest here.

We used this approach to analyze the HapMap 2 sample of chromosomes from the Yoruba in Ibadan, Nigeria (the YRI population) [23]. The results show that *p_D_* ^max^ is highly significant for all 22 autosomes. (The second test statistic, *n*_P_, is found to be sensitive to nuisance parameters used by our method, and so it seems not to be robust for testing patterns of disequilibria.) These results suggest that there are disequilibria extending across large genetic distances in the this population.

This study is certainly not the first to report disequilibria between distant pairs of chromosomal regions in humans. A number of studies have reported disequilibria between regions separated by more than 1 cM (e.g. [38]–[40]). To our knowledge, however, these previous reports primarily involve populations with histories of nonequilibrium demography (such as bottlenecks and/or colonization) that can generate disequilibria [1]. It is perhaps more surprising to detect LRLD in an African population that has not had the genetic perturbations experienced by populations that expanded out of Africa.

What processes might be responsible for the LRLD in the YRI? The Introduction gives six possibilities. One hypothesis is that the sample includes some sort of structure. We considered the effects of the three pairs of relatives that are included in the YRI sample, but found that the patterns of LRLD for Chromosome 1 are largely insensitive to whether or not those relatives are excluded. Population admixture, which is another type of structure, was not detected in the original publication on the HapMap dataset [23].

A second hypothesis is that LRLD results from random genetic drift, perhaps amplified by recent changes in the demography of the YRI population. This is perhaps the most plausible explanation, but it is difficult to test. Simulating datasets comparable to what we analyzed is computationally hard, even with state-of-the-art algorithms [41]. Even more problematic is that simulated datasets would have to account for the demographic history of the population, and those would need to be estimated using the very patterns that we are trying to explain. That is, it seems likely that a neutral model could be fit to the data, but that would not rule out alternative hypotheses.

Epistatic selection is another explanation for these patterns. We do not find a significant correlation between patches of LRLD and regions identified as targets of selection in a recent review [36]. That conclusion, however, rests on correcting the significance level for the very large comparisons done, so it is possible that there is an association that we missed because of a conservative statistical criterion. A useful way to exploit the patches that we identified is as candidates for epistatic selection.

Chromosome inversions that are segregating in the population can also cause LRLD at map lengths that correspond to the size of the inversions [16]. Bansal et al. [14] used this fact to develop an algorithm that can detect inversions using resequencing when the inversions are at high frequency, and used it to identify 71 candidate inversions in the YRI population. It seems unlikely, however, that inversions explain much of the LRLD we observed. None of the breakpoints of those inversions fall within 0.25 cM of a patch of LRLD that we identified. Further, only 7 patches have sites near the breakpoints of any known human inversion [37].

A final possible explanation for the patterns of LRLD that we see is that they are artifacts in the data. Several types of errors (including miscalled SNPs and phasing errors) will tend to obscure disequilibria, and so the significant LRLD we find does not result from them. Other kinds of errors, however, could generate correlations between distant pairs of SNPs. For example, when there are gene duplications on the same chromosome of the reference genome (either real or the result of assembly error), different reads from the same locus will be assigned to each of the duplicates, leading to the appearance of LRLD. This possibility could be explored by determining if patches of high LRLD identified here correlated with pairs of gene duplicates.

Ultimately, the cause of the LRLD reported cannot be determined by our approach. The statistical and computational methods proposed here serve as exploratory tools that allow one to detect disequilibria on a genetic scale that was previously inaccessible. Using these methods on other data sets and uncovering the causes of the patterns that they reveal are exciting challenges for future work.
